# Modeling the covariates effects on the hazard function by piecewise exponential artificial neural networks: an application to a controlled clinical trial on renal carcinoma

**DOI:** 10.1186/s12859-018-2179-1

**Published:** 2018-07-09

**Authors:** Marco Fornili, Patrizia Boracchi, Federico Ambrogi, Elia Biganzoli

**Affiliations:** 1 Department of Clinical Sciences and Community Health, University of Milan, via Venezian 1, 20133 Milan, Italy; 2 Unit of Medical Statistics, Biometry and Bioinformatics, Fondazione IRCSS Istituto Nazionale dei Tumori, via Venezian 1, 20133 Milan, Italy

**Keywords:** Hazard function, Neural networks, Piecewise exponential model, Survival analysis

## Abstract

**Background:**

In exploring the time course of a disease to support or generate biological hypotheses, the shape of the hazard function provides relevant information. For long follow-ups the shape of hazard function may be complex, with the presence of multiple peaks. In this paper we present the use of a neural network extension of the piecewise exponential model to study the shape of the hazard function in time in dependence of covariates. The technique is applied to a dataset of 247 renal cell carcinoma patients from a randomized clinical trial.

**Results:**

An interaction effect of treatment with number of metastatic lymph nodes but not with pathologic T-stage is highlighted.

**Conclusions:**

Piecewise Exponential Artificial Neural Networks demonstrate a clinically useful and flexible tool in assessing interaction or time-dependent effects of the prognostic factors on the hazard function.

## Background

Many analyses of survival data in controlled clinical trials aim to study the effect of therapeutic strategies accounting for prognostic factors, leaving the shape of the baseline hazard function undefined (Cox model). On the other hand the pattern of the hazard function, describing the disease dynamics, may provide a relevant role for a deeper insight of the treatment effect during time suggesting or supporting clinical hypotheses. When long follow-up is available, the shape of hazard function may be complex, for example with a multi-peak pattern.

In addition to the adjusted analysis of treatment effect, it is usual to perform an exploratory evaluation of treatments effects in subgroups of patients characterized by combinations of covariate categories. The aim is to suggest a possible better targeting of the therapeutic strategies to be confirmed by further studies. From the modeling point of view, interactions of treatment and covariates are needed. For a deeper investigation some high order interactions could be considered which are difficult to be modeled explicitly. Accordingly, sufficiently flexible techniques are needed for an adequate estimation. One of the proposed approaches is the GLM implementation of the piecewise exponential [1], where the smoothing of the hazard function is performed by splines.

In this paper we illustrate the use of the extension of the piecewise exponential model by neural networks, the Piecewise Exponential Artificial Neural Networks (PEANN, [2]). In comparison with the GLM and splines approach, PEANN does not require the explicit modeling of the interaction and/or linearity effects of covariates, which in lack of clinical-biological hypotheses of the phenomenon is a strong advantage. Furthermore, the complex architecture of neural networks, with their theoretically proved approximating properties [3], allows in principle greater flexibility in comparison to splines.

We consider here the application of PEANN to a renal cancer dataset, already examined by Kaplan-Meier estimator and Cox regression model in [4]. To avoid the rigidity of proportional hazard hypothesis of Cox model in studying the effect of covariates and further to obtain the shape of the hazard function, neural network can be a convenient choice. As suggested by the CONSORT Statement [5] for the analysis of randomized clinical trials, inference on treatment effects was performed by 95% confidence intervals.

## Methods

In the piecewise exponential model [6] the time range is subdivided into a number of intervals in which the hazard function is supposed to be constant. By adopting splines, it is possible to smooth the time dependence of the hazard function over the time intervals. Usually Generalized Linear Models (GLMs) with Poisson error are adopted for inference.

We have proposed [2] to model the hazard function *h(t;* **x***)* as a function of time and the covariates vector **x** by artificial neural networks, which represent a flexible tool successfully adopted to flexibly model the dependence on continuous and categorical covariates.

The hazard function is modeled by means of the following feed-forward artificial neural network:

$$ h\left(t;\mathbf{x}\right)=\exp \left({\beta}_0^{(2)}+\sum \limits_{k=1}^H{z}_k{\beta}_k^{(2)}\right), $$

where

$$ {z}_k=\mathrm{logis}\left({\beta}_{0k}^{(1)}+\sum \limits_{l=1}^p{x}_l{\beta}_{lk}^{(1)}+t{\beta}_{p+1,k}^{(1)}\right), $$

*exp* and *logis* stand for the exponential and the logistic function respectively, and the *β’*s represent unknown coefficients.

This net is composed of three layers: an input layer with *p* covariates *x*_*l*_ and *t*, an intermediate layer with *H* hidden units *z*_*k*_ and finally the single output unit *h*. Moreover a constant unit set to *1* “feeds” every non-input unit.

To estimate the coefficients *β*’s the following error function is minimized:

$$ E=-\log L+\lambda \left(\sum \limits_{k=0}^H\sum \limits_{l=0}^{p+1}{\beta}_{lk}^{(1)2}+\sum \limits_{k=0}^H{\beta}_k^{(2)2}\right), $$

where the first term is the negative log-likelihood and the second term is a quadratic penalty [3]. The likelihood for *N* subjects is [6]:

$$ L=\prod \limits_{i=1}^N\prod \limits_{j=1}^{J_i}\frac{h{\left({t}_j;{\mathbf{x}}_{\mathbf{i}}\right)}^{d_{ij}}}{\exp \left(h\left({t}_j;{\mathbf{x}}_{\mathbf{i}}\right){\tau}_{ij}\right)}, $$

where *d*_*ij*_ *= 1* if the *i*-th subject has the event in the *j*-th interval and *0* otherwise, *τ*_*ij*_ is the follow-up time for the *i*-th subject in the *j*-th interval, *J*_*i*_ the index of the last interval in which the *i*-th subject is still under observation, **x**_**i**_ the covariate vector for the *i*-th subject, and *t*_*j*_ the midpoint of the *j*-th interval.

The effect of the penalization is that of increasing the performance of the optimization routines and of preventing over fitting. The decay parameter λ controls the trade-off between smoothness and fitting to the data. Minimization of the error function was performed by the quasi-Newtonian algorithm BFGS with analytical gradient.

The flexibility of the neural networks depends on both the number of hidden units and the decay parameter. One strategy to select the model is to recur to cross-validation. We randomly assigned the subjects to one of ten subsets of about the same size (tenfold cross-validation). Excluding each subset at a time, ten nets were fitted on the remaining nine subsets; each net was initialized with different starting values for the parameters. The following criterion was thus computed:

$$ CV=\frac{1}{\sum \limits_{i=1}^N{J}_i}\sum \limits_{i=1}^N\sum \limits_{j=1}^{J_i}\left({d}_{ij}\log {\widehat{h}}^{\left(-m(i)\right)}\left({t}_j;{\mathbf{x}}_{\mathbf{i}}\right)-{\widehat{h}}^{\left(-m(i)\right)}\Big({t}_j;{\mathbf{x}}_{\mathbf{i}}\Big){\tau}_{ij}\right), $$

where ${\widehat{h}}^{\left(-m(i)\right)}$ indicates the average over the ten hazard functions estimated excluding the subset *m(i)* in which the *i*-th subject falls. Based on Bayesian consideration [3], it is suggested for the decay parameter an explorative range of 10^− 3^ - 10^− 1^. In performing both cross validation and estimations, the averaging over fits obtained from different initial values for the parameters has been suggested [7].

For inference confidence intervals can be based on nonparametric bootstrap. To consider inference on covariate effects, percentiles confidence intervals for log hazard ratios have been obtained using 2000 bootstrap samples, conditional to the number of hidden units and the decay parameter value selected from cross validation. The confidence intervals were computes as follows: for each of 2000 bootstrap samples, an hazard estimate was computed as the average over ten nets initialized with the final coefficient values of previous fitted nets used for estimation; the 2.5 and 97.5 percentiles of the distribution of the 2000 hazard estimates were pointwise computed with bias correction [8].

For an application, we considered a data set [4] on a randomized controlled clinical trial comparing adjuvant interferon alpha-2b and simple observation without interferon in patients with Robson stages II and III renal cell carcinoma. Time from radical nephrectomy to the occurrence of local or distant metastasis as first event (event-free survival) was considered. The median follow-up was 62 months. Among the 123 treated patients there were 51 relapses, while among the 124 control patients 38 relapses occurred. In the analysis, besides time and treatment, the following covariates were considered: tumor grade, categorized as G1-G2 or G3-G4; pathologic T stage, categorized as pT2, pT3a or pT3b; metastatic lymph nodes, categorized as pN0, pN1 or pN2-pN3. For PEANN follow-up was truncated at 72 months and time range was subdivided into three months intervals. The 95% pointwise confidence intervals were calculated on the log hazard ratio of interferon versus controls.

To describe the relationship between the PEANN-estimated cumulative incidence at 6 years and the covariates, we have resorted to CART regression tree [9]. As results of the tree in terms of log cumulative hazard were superimposable to those obtained in terms of event cumulative incidence at six years we report the latter results because of their better interpretability.

All the routines for the analysis were written in R v.3.3.2 language.

## Results

To select the structure of the network, a ten-fold cross validation was applied considering 3, 6 and 9 hidden units and penalty coefficients 0.001, 0.005, 0.02 and 0.1. The scores of the cross validation criterion (Table 1) show a better performance in correspondence to 3 hidden units and a decay parameter equal to 0.1, which we therefore used.

**Table 1:** Cross validation scores. Ten-fold cross-validation scores obtained by averaging over ten fits for various values of the number of hidden units (H) and of the decay parameter

Decay parameter	H = 3	H = 6	H = 9
0.001	0.1595	0.3089·10^3^	0.6267·10^6^
0.005	0.1601	0.3324	0.1205·10^3^
0.02	0.1601	0.1725	0.2081
0.1	0.1589	0.1596	0.1596

**Fig. 1:**
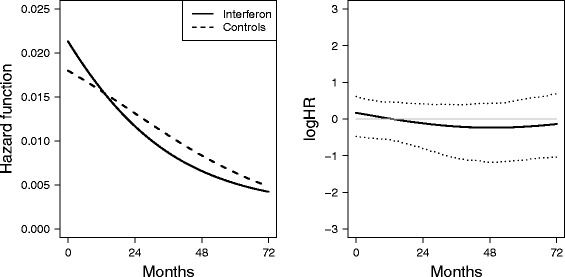
Hazards and log hazard ratio for the two treatments. Left panel: Adjusted hazard function estimates for interferon (solid lines) and control (dashed lines). Right panel: Adjusted log hazard ratio (solid) with 95% bootstrap confidence intervals (dotted). The reference log HR = 0 (thin grey line) is also reported

We then considered a neural network with time, treatment, tumor grade, pathologic T stage and metastatic lymph nodes using the average over the ten fits obtained from different initial choice of parameters. Figure 1 illustrates the adjusted hazard function estimates for interferon and controls.

Adjusted hazard functions are calculated as weighted average of the individual estimates, with weights proportional to the number of individuals at each level of covariates [10]. The trend of the hazard function for both groups shows a monotone decrease. The 95% bootstrap confidence interval of the log hazard ratio in time is in agreement with the non-significance of the difference between the two treatment groups according to the log-rank test observed in [4].

**Fig. 2:**
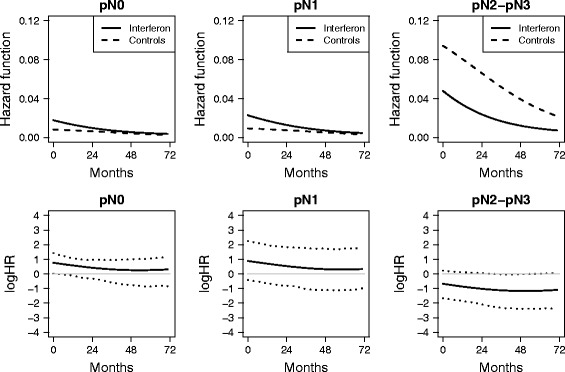
Hazards and log hazard ratios for the two treatments by lymph nodes. Top panels: Adjusted hazard function estimates for interferon (solid lines) and control (dashed lines) groups for the three lymph nodes categories. Bottom panels: Adjusted log hazard ratio (solid lines) with 95% bootstrap confidence intervals (dotted lines). The reference log HR = 0 (thin grey line) is also reported

To investigate the possible interaction between treatment and lymph nodes, highlighted in [4], the average estimated hazard for these subgroups have been obtained (Fig. 2).

The fitted curves seem to agree with the interaction, with a protective role of interferon in the pN2-pN3 category. Based on the bootstrap confidence intervals, there is evidence of a harmful effect of interferon in patients with pN0 stage at the beginning of the follow-up time till about 24 months while, on the contrary, a potential protective effect appears to be present for patients with pN2-pN3 tumors.

**Fig. 3:**
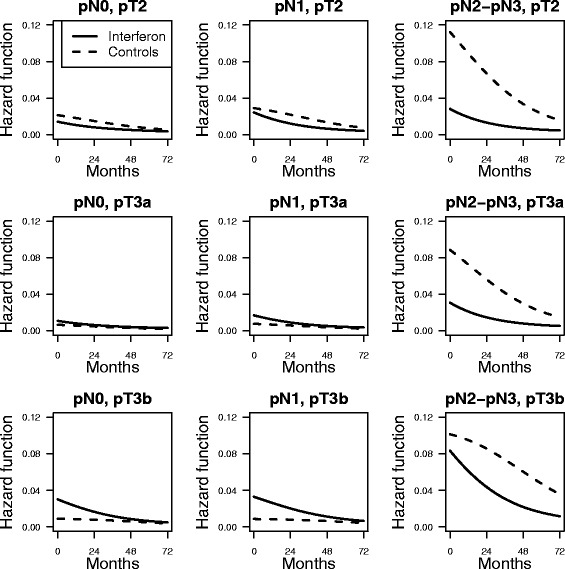
Hazards for the two treatments by lymph nodes and pathologic T stage. Adjusted hazard function estimates for interferon (solid lines) and control (dashed lines) groups cross-classifying by lymph nodes and pathologic T stage

**Fig. 4:**
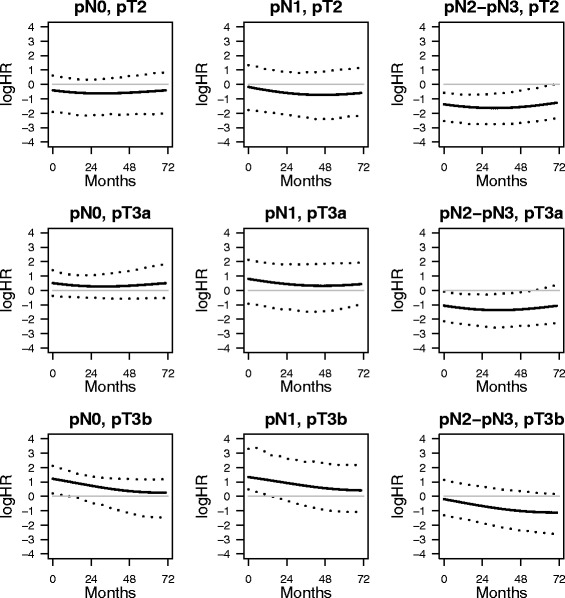
Log hazard ratios for the two treatments by lymph nodes and pathologic T stage. Adjusted log hazard ratios estimates for interferon (solid lines) and control (dashed lines) cross-classifying by lymph nodes and pathologic T stage. The reference log HR = 0 (thin grey line) is also reported.

Interactions among treatment, lymph nodes and pathological T stage are explored by multipanel conditioning plots of the hazard functions (Fig. 3) and of the log hazard ratios of the interferon vs. the control group (Fig. 4).

While it was evident the interaction with pN, an interaction between pT and pN is not apparent. Based on confidence intervals, it can be observed a significant harmful effect of interferon before 24 months for patient with pN0 and pT3b tumors. On the contrary, the protective effect of interferon is significant over the entire follow-up time for patients with pN2-pN3 and with either pT2 or pT3a tumors.

**Fig. 5:**
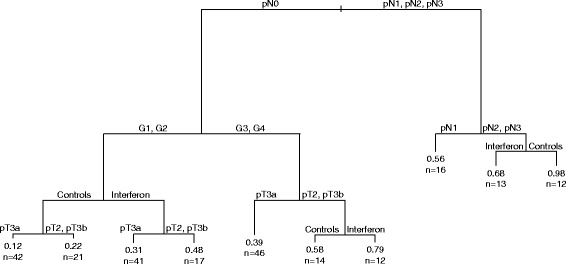
Regression tree. Regression tree of the event cumulative incidence at six years estimated by PEANN as a function of the considered covariates. Each node is labeled with a binary question about a covariate. In the terminal nodes the number of patients and the estimated incidence are reported

As regards the relationship between the PEANN-estimated six years cumulative incidence and clinical characteristics, regression tree structure (Fig. 5) suggests the presence of complex interactions patterns among treatment and covariates. In particular, in patients with pN2-pN3 interferon shows a lower incidence with respect to controls. An opposite behavior is shown for those pN0 patients who have G1-G2 or G3-G4 and pT2 or pT3b.

## Discussion

The shape of the hazard function may be of great clinical interest to support clinical-biological hypotheses or to plan follow-up visits. As the number of prognostic factors considered is ever increasing, statistical approaches able to model non-linear, non-additive and time-dependent effects of the covariates on the hazard function should be investigated. We proposed to recur to neural networks as flexible non-linear extension of GLMs for survival data [11, 12]. Even in controlled clinical trials, for which classical survival regression techniques are routinely applied for treatment evaluation, neural networks can be useful to explore potential complex interactions between treatment and covariates, aiming to suggest clinical targeting hypotheses.

A critical aspect is the representation of model results. We resorted to multipanel conditioning plots to evaluate the possible different behavior of the hazard function and hazard ratio in different combinations of variables categories. Though, this approach does not allow the assessment of treatment effect for more than two covariates at a time. A possibility is to resort to a “rule-extraction” approach that we simplified using a regression tree.

The present application allowed to investigate second order interaction with a time-dependent effect, which was not considered in the previous Cox regression analysis because of its complexity [4]. Although the confidence intervals obtained are quite wide due to the limited sample size, a qualitative interaction was observed with different treatment effects in pN/pT subgroups of patients. It must be taken in account that the nonparametric bootstrap confidence intervals that we adopted do not make distributional assumptions but are not the most efficient ones. Possibly more suitable approaches useful for neural networks could be investigated. The regression tree was used only for exploratory aims and a complex interaction pattern is suggested by the results. For inference purposes confidence intervals should be calculated for the cumulative incidence in each leaf of the regression tree. Nevertheless even larger confidence intervals are expected because of the limited number of patients for each terminal node.

The smoothing of the shape of the hazard function in dependence on treatment and covariates can be useful to understand the mode of action of the treatments. Neural networks could be also associated to a classical analysis as an exploratory tool. The traditional use of neural networks is only for exploratory purposes without the application of inferential procedures. The advantages of neural networks is mainly linked to the flexibility of the predictor that does not need the explicit joint modeling of the effect of covariates, which could be complex and unpredictable in the absence of prior knowledge. Recent developments suggest using bootstrap to obtain confidence intervals, providing further appeal to this technique.

## Conclusions

Artificial neural networks are not usually adopted to analyze data from clinical trials where survival analysis is needed. Nonetheless PEANN, flexibly modeling the shape of the hazard function, allows to investigate time-dependent treatment effectsin subsets corresponding to covariates combinations without prior specification of the model predictor structure. The application to a randomized controlled trial on renal carcinoma has shown how PEANN, together with regression trees, permits the identification of potential interactions to be explored by further studies on the differential treatment effect as a function of patient clinico-pathological characteristics.
